# Zika Virus Infection Induces Acute Kidney Injury Through Activating NLRP3 Inflammasome Via Suppressing Bcl-2

**DOI:** 10.3389/fimmu.2019.01925

**Published:** 2019-08-14

**Authors:** Ting Liu, Lantian Tang, Hui Tang, Jieying Pu, Sitang Gong, Danyun Fang, Hui Zhang, Yi-Ping Li, Xun Zhu, Weidong Wang, Minhao Wu, Yuhui Liao, Chunling Li, Haibo Zhou, Xi Huang

**Affiliations:** ^1^Program of Infection and Immunity, The Fifth Affiliated Hospital of Sun Yat-sen University, Zhongshan School of Medicine, Sun Yat-sen University, Zhuhai, China; ^2^Program of Immunology, Department of Internal Medicine and Guangzhou Institute of Pediatrics, Guangzhou Women and Children's Medical Center, Zhongshan School of Medicine, Sun Yat-sen University, Guangzhou, China; ^3^The Sixth Affiliated Hospital of Guangzhou Medical University, Qingyuan, China

**Keywords:** Zika virus, acute kidney injury, aquaporins, NLRP3 inflammasome, Bcl-2

## Abstract

Zika virus (ZIKV) is a newly emerging flavivirus that broadly exhibits in various bodily tissues and fluids, especially in the brain, and ZIKV infection often causes microcephaly. Previous studies have been reported that ZIKV can infect renal cells and can be detected in the urine samples of infected individuals. However, whether ZIKV infection causes renal diseases and its pathogenic mechanisms remains unknown. Here, we identified that ZIKV infection resulted in acute kidney injury (AKI) in both newborn and adult mouse models by increasing the levels of AKI-related biomarkers [e.g., serum creatinine (Scr), kidney injury molecular−1 (Kim-1), and neutrophil gelatinase-associated lipocalin (NGAL)]. ZIKV infection triggered the inflammatory response and renal cell injury by activating Nod-like receptor 3 (NLRP3) inflammasome and secreting interleukin-1β (IL-1β). IL-1β inhibited aquaporins expression and led to water re-absorption disorder. Furthermore, ZIKV infection induced a decreased expression of B-cell lymphoma-2 (Bcl-2) in the kidney. Overexpression of Bcl-2 attenuated ZIKV-induced NLRP3 inflammasome activation in renal cells and down-regulated PARP/caspase-3-mediated renal apoptosis. Overall, our findings demonstrated that ZIKV infection induced AKI by activating NLRP3 inflammasome and apoptosis through suppressing Bcl-2 expression, which provided potential therapeutic targets for ZIKV-associated renal diseases.

## Introduction

Zika virus (ZIKV) is a mosquito-borne virus, a member of the *Flaviviridae* family and ZIKV infection generally causes mild clinical symptoms, such as fever, rash, muscle pain, and conjunctivitis (1). However, current epidemic of ZIKV infection has been reported to cause severe diseases, including microcephaly in newborns and Guillain-Barré syndrome in adults (2, 3). ZIKV preferentially infects neural progenitor cells, mature neurons and astrocytes to impair fetal brain development and cause microcephaly. ZIKV can also infect other organs, such as eyes, testis, placenta, uterus, and vagina, resulting in viral detection in multiple bodily fluids, including tears, saliva, semen, cervical mucus, and urine (4, 5). Thus, recent studies on ZIKV pathogenesis have demonstrated that ZIKV exhibits a broad tissue tropism and has the capacity to cause severe diseases (6).

Most clinical studies have demonstrated that high level of ZIKV viral loads can be detected in the urine samples of both adult and infant patients (7, 8). Previous studies show that ZIKV can infect glomerular parenchymal cells and renal proximal tubular epithelial cells, which may serve as potential reservoirs for ZIKV in kidney (9, 10). However, whether ZIKV infection causes renal disease remains unknown. Cases are reported that two pediatric patients are diagnosed with idiopathic nephrotic syndrome after ZIKV infection and they are in complete remission of the disease after treatment (11, 12). But the length of hospital stay duration is too short to allow further observation of the sequelae. Thus, the pathogenesis and long-term consequences of ZIKV infection in the kidney still need to be studied.

Virus infection has been found as an important causative agent of acute kidney injury (AKI) (13–15), which can be triggered by hyperactivated Nod-like receptor protein 3 (NLRP3) inflammasome (16, 17). NLRP3 inflammasome is composed of NLRP3, apoptotic speck protein containing a caspase recruitment domain (ASC) and pro-caspase-1 (18). Upon activation, NLRP3 interacts with ASC, which recruits caspase-1 and induces its activation. Then the activated caspase-1 processes pro-IL-1β into its mature form IL-1β, thus triggering inflammatory response and tissue damage (19, 20). Particularly, the increased IL-1β can down-regulate aquaporin 2 (AQP2) expression in collecting duct cells, which contributes to the impairment of reabsorption of water (21). Studies have reported a greatly increased expression levels of IL-1β and IL-18 in the serum of patients infected with ZIKV, indicating that inflammasomes may be activated by ZIKV infection and involved in tissue damage (22). However, whether NLRP3 inflammasome participates in the kidney injury induced by ZIKV infection and its underlying mechanism remain unknown. Recent studies report that Bcl-2 is a novel negative regulator of inflammasome activation, which can suppress the activation of caspase-1 by directly binding to NLRP1, thus attenuating the cleavage of IL-1β (23–25). Even though the role of Bcl-2 in NLRP1 inflammasome activation mechanism is well-appreciated, it's still unknown whether Bcl-2 is involved in regulating NLRP3 inflammasome activation induced by ZIKV infection.

In the present study, ZIKV showed high aggressiveness to renal epithelial cells and ZIKV infection induced AKI in both newborn and adult mice, which was associated with tubular injury, uncontrolled inflammation and renal cell apoptosis. ZIKV infection activated NLRP3 inflammasome and triggered the production of IL-1β in the kidney, which directly decreased the expression of aquaporins, thus leading to the water re-absorption disorder. More importantly, our study identified Bcl-2 as a negative regulator of ZIKV-induced NLRP3 inflammasome activation and renal cell apoptosis, which provided a new insight of the therapeutic target for renal diseases induced by viral infection.

## Materials and Methods

### Virus and Cell Infection

The ZIKV (ZG-01) stocks used in the present study were isolated from the urine of a Chinese patient returning from Venezuela in February 2016. The urine samples were removed endotoxin and subsequently inoculated into a confluent monolayer of Vero cells; the stocks were stored in aliquots at −80°C until use. The virus stocks were titrated by standard plaque assays (PFU assay) using Vero cells. The human proximal tubular cells (HK-2) were provided by Professor Weidong Wang, culturing in DMEM/F12 (1:1, Gibco) supplemented with 10% FBS, 100 IU/mL of penicillin and 100 μg/mL of streptomycin and were maintained at 37°C in a fully humidified atmosphere with 5% CO_2_. The primary murine renal epithelial cells isolated from the kidneys of 7 day-old newborn mice. All cells were trypsinized and seeded on 24-well plates at a density of 1 × 10^5^ cells per well. The cells inoculated with ZIKV (MOI = 2) for 1 h at 37°C and washed with phosphate-buffered saline (PBS), followed by culturing with complete medium for 24 h. To inhibit NLRP3 activation, cells were pretreated with 10 μM MCC950 (S7809, Selleck, CN) for 30 min. The cells were infected with ZIKV in the presence of MCC950 or vehicle control for 1 h at 37°C, washed with PBS, and cultured with medium containing MCC950 or vehicle control for 24 h.

### Animal Models and Experiments

All mice were purchased and maintained under specific pathogen-free conditions at the research animal facility of Sun Yat-sen University. For the newborn mouse model, one- to 2 day-old BALB/c mice were intraperitoneally (i.p.) injected with 10^5^ PFU ZIKV in a 50 μl volume and monitored over 15 days. An equal amount of vehicle PBS was i.p. injected into the uninfected mice (mock group) (Supplementary Figures 1A–D). To inhibit NLRP3 and IL-1β activation, mice were i.p. injected with 10 μg MCC950 per gram body weight (BW) or 35 μg/g BW Anakinra (143090-92-0, ChemLeader, CN) or PBS (vehicle control) ever 2 days beginning from 2 days post-infection until tissue harvest. For the adult mouse model, an IFNαR1-blocking mouse antibody (MAR1-5A3, BioXcell, GER) or its isotype control mouse antibody (GIR-208, BioXcell, GER) (2 mg per mice) were i.p. injected into wild-type C57BL/6 mice at the day before infection (26). Then the mice were i.p. injected with ZIKV (1 × 10^5^ PFU) in a 200 μl volume and treated with MCC950 (10 μg/g BW) or Anakinra (75 μg/g BW) or PBS (vehicle control) daily beginning from the day post-infection until tissue harvest. All experiments were performed in accordance with the National Institutes of Health Guide for the Care and Use of Laboratory Animals, and the study was approved by the animal Ethics Committee of Zhongshan School of Medicine, Sun Yat-sen University (ethics reference number: 2016-158). All experiments were operated in labs of BSL2 to ensure safety.

### Quantitative RT-PCR (qRT-PCR)

The tissues of experimental mice were harvested, weighed, and homogenized with zirconia beads in 1 ml of PBS using the Tissue Lyser II instrument (QIAGEN, Frankfurt, GER). Total RNA was isolated with Trizol reagent (Invitrogen, Carlsbad, CA, USA) according to the manufacturer's instructions. For urine and serum detection, the Log Pure Viral DNA/RNA Kit (serum/urine) (Magen, Guangzhou, CN) was used to extract the ZIKV RNA. First-strand cDNA synthesis was performed using the Revert Aid First Strand cDNA Synthesis Kit (Thermo Fisher Scientific, Waltham, MA, USA). Quantitative real-time PCR detection of RNA copies were performed on the Bio-Rad CFX96 real-time analysis system using Taq Man Master Mix. Viral loads were expressed on a log10 scale as viral RNA equivalents per milligram or per milliliter upon comparing with a standard curve produced by gradient 10-fold dilutions of ZIKV RNA. To detect ZIKV RNA, a primer set previously reported by Lanciotti and coworkers was used (27). The mRNA expression levels of AKI-related biomarkers (Kim-1, NGAL), inflammatory factors were normalized to β-actin expression. The primer sequences used are shown in Table 1.

**Table 1:** Primer sequences for real-time PCR (“m”:mouse; “h”:human; “Fwd”:Forward; “Rev”:Reverse).

**Gene**	**Primer Sequence (5′−3′)**
mKim-1	Fwd: ACATATCGTGGAATCACAACGAC
	Rev: ACAAGCAGAAGATGGGCATTG
hKim-1	Fwd: CTG CAG GGA GCA ATA AGG AG
	Rev: ACC CAA AAG AGC AAG AAG CA
mNGAL	Fwd: TGGCCCTGAGTGTCATGTG
	Rev: CTCTTGTAGCTCATAGATGGTGC
hNGAL	Fwd: GGGAAGTGGTATGTGGTAGG
	Rev: AGGGAAGACGATGTGGTTT
mNLRP1	Fwd: ACCGAGTTCAGTTACCCA
	Rev: TGTCAAACAGAGGTCCAAC
mNLRP3	Fwd: CGAGACCTCTGGGAAAAAGCT
	Rev: GCATACCATAGAGGAATGTGATGTACA
hNLRP3	Fwd: TGAACAGCCACCTCACTT
	Rev: CAACCACAATCTCCGAAT
mAIM2	Fwd: CACCCTCATGGACCTACACTACCG
	Rev: CCATAGGGGCTGCTCGATCCAC
mNLRC4	Fwd: CTCTCATGGTGGAAGCCAGTCC
	Rev: GACAGAGACTTGACTATGTAATCC
hIL-18	Fwd: GCGTCACTACACTCAGCTAAT
	Rev: GCGTCACTACACTCAGCTAAT
mIL-18	Fwd: GTGAACCCCAGACCAGACTG
	Rev: CCTGGAACACGTTTCTGAAAGA
mIL-1β	Fwd: GAAATGCCACCTTTTGACAGTG
	Rev: TGGATGCTCTCATCAGGACAG
mIL-6	Fwd: TCTATACCACTTCACAAGTCGGA
	Rev: GAATTGCCATTGCACAACTCTTT
TNF-α	Fwd: CTGAACTTCGGGGTGATCGG
	Rev: GGCTTGTCACTCGAATTTTGAGA
TGF-β	Fwd: GCAACATGTGGAACTCTACCAGA
	Rev: GACGTCAAAAGACAGCCACTCA
MCP-1	Fwd: TTAAAAACCTGGATCGGAACCAA
	Rev: GCATTAGCTTCAGATTTACGGGT
ZIKV-E	Fwd: CCGCTGCCCAACACAAG
	Rev: CCACTAACGTTCTTTTGCAGACAT
	Probe:AGCCTACCTTGACAAGCAGTCAGACACTCAA
mGAPDH	Fwd: TCATGGGTGTGAACCATGAG
	Rev: CTAAGCAGTTGGTGGTGCAG
	Probe: ATGACAACAGCCTCAAGATCATCAGCAATG

### *In situ* Hybridization (ISH)

The RNA ISH of ZIKV was performed using the indicated riboprobes that target multiple genes of ZIKV (Advanced Cell Diagnostics, Newark, CA, USA) according to the manufacturer's instructions. In brief, the kidney sections were dewaxed by rinsing with xylene, 100% ethanol, 95% ethanol, 70% ethanol, and deionized water twice every 5 min. Then the RNA of ZIKV was detected using the indicated RNA probe from Advanced Cell Diagnostics (Cat No. 467771). Hybridization signals were visualized by chromogenic reactions using DAB chromogen, followed by redyeing with hematoxylin. Images of stained tissues were visualized and captured using an Olympus fluorescence microscope.

### Immunofluorescence Assay

For cells *in vitro*, HK-2 and primary kidney cells were seeded onto the slides of 24-well plates at a density of 7 × 10^4^ cells per well-followed by ZIKV infection. The cells were fixed with 4% paraformaldehyde for 30 min at room temperature and then permeabilized with 0.1% Triton X-100 in PBS. Then the cells were incubated with anti-ZIKV-E protein antibody (clone D1-4G2-4-15, Millipore, GER) overnight. After washing three times with PBS, the cells were incubated with secondary anti-mouse antibody Zymosan Alexa Fluor 488 Fluorescent Bioparticles (REF Z23373, Thermo Fisher Scientific, USA) for 2 h. Finally, cells nuclei were stained by DAPI (REF D1306, Thermo Fisher Scientific, USA).

For kidney sections, the samples were dewaxed according to previous procedures (as ISH described). The kidney slides containing with citric acid buffer (PH = 6.2) were performed in microwave for antigen retrieval and blocked with 5% goat serum in PBS. Then, the kidney sections were incubated with anti-ZIKV-E protein antibody in 4°C overnight. Afterward, the sections were incubated with the indicated secondary anti-mouse antibody for 2 h, and cell nuclei were stained with DAPI.

### Hematoxylin and Eosin (H & E) Staining

For histological analysis, kidneys fixed in 4% paraformaldehyde, embedded in paraffin and sectioned at a 5 μm thickness were stained with H&E. Images of stained tissues were visualized and captured using an Olympus fluorescence microscope. The kidney histological scores were quantified in a blinded fashion. Renal tissue injury was assessed as follows: tubular dilatation and vacuolization, interstitial edema, brush border defect and inflammatory cell infiltration.

### Collagen Hybridizing Peptide (CHP) Staining

The kidney slides were dewaxed according to previous procedures (as ISH described), and the deparaffinized tissue stained directly without any antigen-retrieval process. Then the endogenous biotin-blocking kit was used to block any endogenous biotin in the tissue following the manufacturer's protocol. After blocking, the heat-activated single-strand CHPs solutions was prepared according to the reference (28), and the pre-prepared B-CHP (biotin conjugate CHP) solution was pipetted rapidly to each slide within 3 min. Then, the kidney slides were incubated in 4°C in a moisturizing box overnight. After staining, the slides were washed with PBS at room temperature for 5 min to remove the unbound CHP. The kidney sections were subsequently incubated with 10 μM QDs-labeled streptavidin (Vector, US, QS605) in PBS solution. Finally, the kidney sections were washed three times with PBS. In the present study, B-CHP was provided by Professor Yang Li, Department of Pharmaceutics and Pharmaceutical Chemistry, University of Utah.

### Serum Creatinine (Src) Measurement

For serum creatinine measurements, mice were sacrificed, and blood samples were collected from the aorta. The blood was centrifuged, and serum was separated, aliquoted and stored at −80°C until use. The concentrations of serum creatinine were measured by an enzyme immunoassay (EIA) kit according to the manufacturer's instructions (BioAssay Systems, Hayward, CA, USA).

### Immunohistochemistry

Kidneys were fixed with 4% paraformaldehyde. Approximately 5 μm-thick slices of kidney sections were stained for AQP1 and AQP2. Briefly, after dewaxing and rehydration according to previous procedures (as ISH described), the kidney slides containing with citric acid buffer (PH = 6.2) were performed in microwave for antigen retrieval and blocked with 5% goat serum in PBS. The kidney sections were incubated with the antibody of AQP1 (ab15080, Abcam, UK) and AQP2 (ab199975, Abcam, UK) in 4°C overnight. After washing with PBS three times, the kidney slides were incubated with biotinylated secondary antibody (Vector Laboratories, USA) for 2 h. Hybridization signals were observed by 30-diaminobenzidine tetrahydrochloride. Sections were finally stained with Mayer's hematoxylin to reveal the nuclei. Microscopic examination was detected with an Olympus microscope.

### Western Blot Analysis

Western blot analysis, 30 μg aliquots of proteins from kidney tissues and cultured cell lysates were separated on an 8–12% SDS-PAGE, transferred to nitrocellulose membranes (Millipore, MA, USA), blocked with 5% BSA in PBS with 0.1% Tween 20 (PBST). The membranes were probed with the following primary antibodies: anti-NLRP3, and anti-caspase-1 (all from Adipogen, CA, USA); anti-IL-1β, anti-ASC, and anti-Bcl-2 (all from Cell Signaling Technology, Danvers, MA); anti-AQP1 and AQP2 (from Abcam, UK). Western blots were performed using a horseradish peroxidase-conjugated secondary antibody, followed by detection with enhanced chemiluminescence. β-Actin was developed as a loading control to normalize the data.

### Urine Output Measurement

Newborn or adult mice were housed in metabolic cages for 6 or 12 h and the urine samples were collected to separate urine tubes. The urine output was measured by microsyringe.

### ELISA

The concentration of IL-1β in the supernatants of mouse kidneys were determined according to the manufacturers' instructions of the ELISA kit from AssayPro (EMI1006-1, USA).

### Overexpression of Bcl-2 in HK-2 Cells

For plasmid construction, the cDNA of Bcl-2 with FLAG-tag was cloned by PCR and inserted into pSG5 vector. The recombinational plasmid was verified by sequencing. HK-2 cells were seeded in 24-well plates at a density of 1 × 10^5^ cells per well. Then, the cells were transfected with the pSG5-FLAG-Bcl-2 recombinational plasmid (2 μg/ml) using lipofectamine 2000 (Invitrogen, Carlsbad, USA) and cultured in 5% CO_2_ incubator at 37°C for 24 h, followed by ZIKV infection. Meanwhile, the MCC950 addition served as positive control to inhibit NLRP3 activation. Briefly, cells were pretreated with MCC950 (10 μM) for 30 min, and then infected with ZIKV (MOI = 2) in the MCC950-containing medium for 1 h at 37°C, washed with PBS and cultured with medium containing MCC950.

### TUNEL Assay

The TUNEL procedure was applied to kidney sections to detect DNA fragmentation as an index of apoptosis. Paraffin sections of 5 μm thickness that were fixed with 4% paraformaldehyde in PBS were stained using an *in situ* TUNEL Detection Kit (Servicebio, Wuhan, CN). Cell nuclei stained with hematoxylin appeared blue, while the nuclei of cells that were positive for apoptosis, developed by the DAB reagent, shown as brownish-yellow. TUNEL-positive cells were counted in 5 randomly selected fields (400 × magnification). Two independent observers blinded to the experimental conditions performed counts and calculated the average number of TUNEL-positive cells. Data were collected from more than 3 independent experiments performed in triplicate.

### Statistics

Statistical analyses were performed using GraphPad Prism 6.0 (GraphPad Inc., La Jolla, CA, USA). For viral burden analysis, the log10 transformed titers were analyzed by Student's *t*-test or the Mann-Whitney test for parametric and non-parametric data. At least three independent experiments were performed in each case, and a *P* < 0.05 was considered statistically significant.

## Results

### ZIKV Infected the Renal Tubular Epithelial Cells

**Figure 1:**
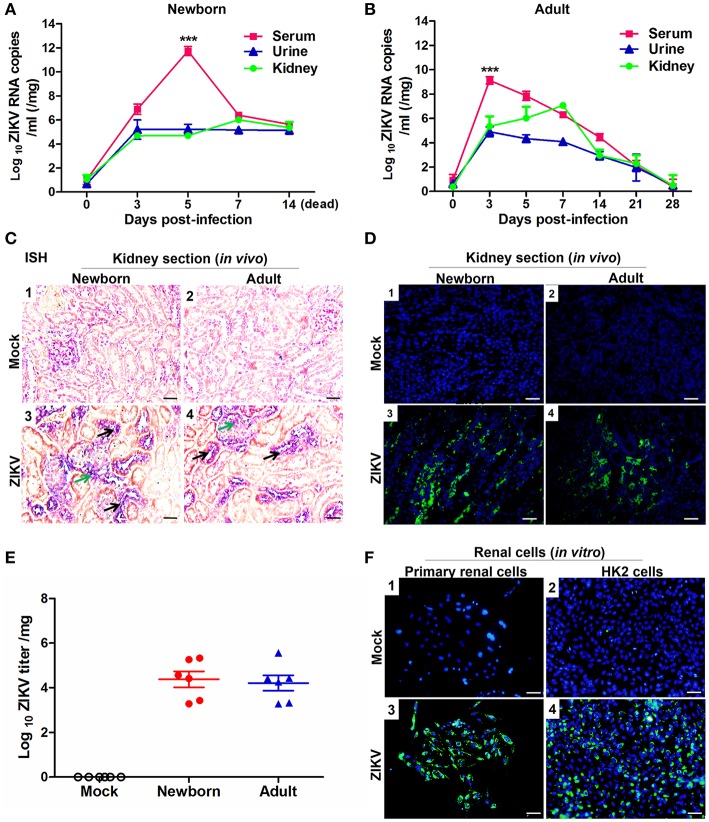
ZIKV infection in the kidneys of mice and *in vitro* renal cells. **(A,B)** Newborn **(A)** and adult **(B)** mice viral loads were measured by qRT-PCR in the serum, urine, and kidney at different time points post-infection. **(C)** Newborn and adult mouse kidney sections were detected with *in situ* hybridization (ISH) using a ZIKV-specific probe. Hybridization signals of ZIKV RNA were developed by a chromogenic reaction and were observed as blue particles, green and black arrows indicated renal glomerular and tubular cells, respectively. Scale bar 20 μm. **(D)** Kidney sections from mock or ZIKV-infected mice were paraffin-embedded and processed, and were stained with antibodies against ZIKV-E protein, ZIKV was detected in green with AlexaFluor^®^488-conjugated secondary antibodies and nuclei was stained with DAPI (in blue). Scale bars, 40 μm. **(E)** Plaque assays for viral titers in the kidneys of newborn and adult mice were performed on 7 days post-infection. **(F)** Mock and ZIKV-infected (MOI = 4) primary renal epithelial cells and human proximal tubular cells (HK-2) cells were cultured for 48 h, stained with DAPI to label nuclei (blue) and an antibody against ZIKV E protein (green), examined by confocal microscopy. Scale bars, 40 μm. Data were presented as mean ± SEM (*n* = 6), ^***^p < 0.001 vs. the viral loads in urine and kidney.

To investigate whether ZIKV infected renal cells and replicated in the kidney, we established ZIKV-infected newborn and adult mouse models corresponding to different age stages, respectively. Then, the viral loads in both serum and urine from infected mice were examined. Newborn mice infected with ZIKV showed the highest viremia level on 5 days post-infection (Figure 1A), while adult mice showed the highest level of viremia on 3 days post-infection (Figure 1B). Although the viremia levels declined, viral RNA was continuously detected in the urine and kidneys of both newborn and adult mice, which suggested ZIKV reproduction in the kidneys. ZIKV RNA presented in the glomeruli and tubules, which was indicated by the green and black arrows, respectively (Figure 1C). The presence of ZIKV in the kidneys was further confirmed by the detection of Immunofluorescence. The pictures showed that ZIKV-E protein (green) expressed around the renal tubule (Figure 1D), indicating that ZIKV may inhabit tubular epithelial cells in the kidneys. In addition, the high viral titers could be detected in the infected kidneys, which confirmed the presence of live ZIKV in the kidneys (Figure 1E). To further clarify the target cells of ZIKV, we examined ZIKV infection in the primary murine renal epithelial cells (Figures 1F1–F3) and the human proximal tubular cells (HK-2) (Figures 1F2–F4). Immunofluorescence staining showed that ZIKV could efficiently infect these renal cells *in vitro* at 48 h post-infection. Thus, our data indicated that the kidney might be a ZIKV reservoir, where the virus probably replicated in the tubular renal epithelial cells.

### ZIKV Infection Induced Acute Kidney Injury in Newborn and Adult Mice

**Figure 2:**
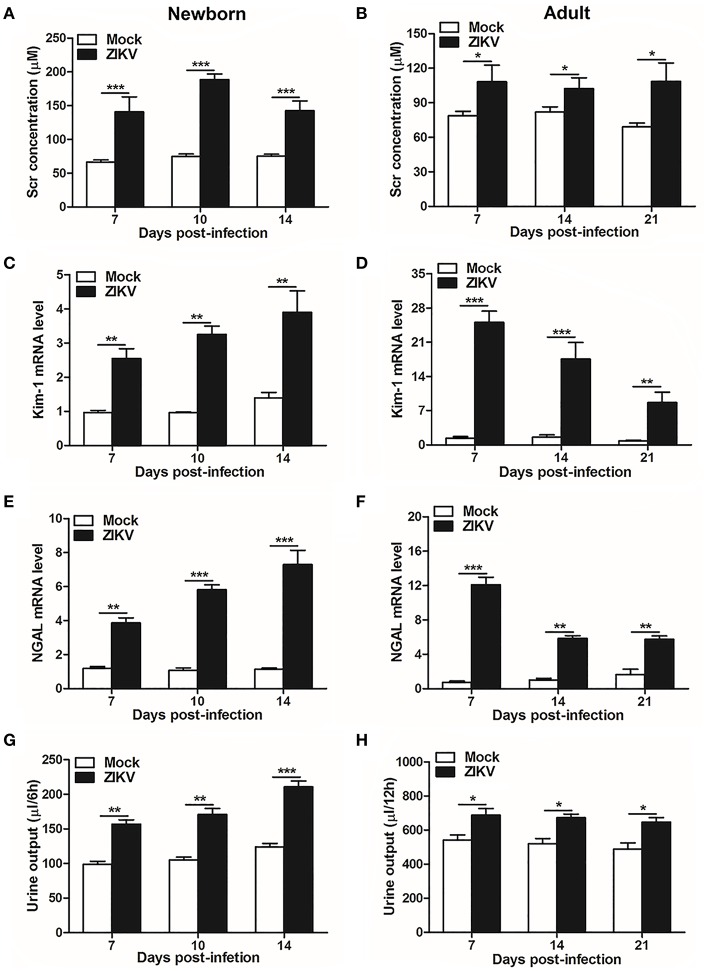
Acute kidney injury in ZIKV-infected newborn and adult mice. **(A,B)** The Serum creatinine (Scr) concentration was tested in newborn mice **(A)** at 7, 10, 14 days post-infection and in adult mice **(B)** at 7, 14, 21 days post-infection using a creatinine assay kit (BioAssay Systems). **(C–F)** The relative mRNA levels of KIM-1 **(C,D)** and NGAL **(E,F)** were determined in the kidneys of newborn mice **(E)** at 7, 10, 14 days post-infection and adult mice **(F)** at 7, 14, 21 days post-infection by qRT-PCR. **(G,H)** Urine output was measured for 6 h in newborn mice **(G)** and 12 h for adult mice **(H)**. Data were presented as mean ± SEM (*n* = 6), ^*^p < 0.05, ^**^p < 0.01, ^***^p < 0.001 vs. mock group.

As renal epithelial cells were permissive to ZIKV infection and replication, next we further investigate whether ZIKV infection affected renal function. Firstly, as the most important biomarker for diagnosing AKI, the serum creatinine (Scr) concentrations were detected in the mouse serum at different time points. Our data showed that Scr concentrations were significantly increased in ZIKV-infected newborn (Figure 2A) and adult mice (Figure 2B) compared to those of mock group, which indicated an impaired glomerular filtration in infected mice. In addition, increased mRNA expression levels of the AKI-related biomarkers Kim-1 and NGAL were detected in ZIKV-infected newborn (Figures 2C,E) and adult (Figures 2D,F) mice, indicating extensive proximal tubular injuries caused by ZIKV infection. Moreover, urine output significantly rose up after ZIKV infection (Figures 2G,H). These results suggested that tubular injuries and water re-absorption disorder occurred in mice infected with ZIKV.

**Figure 3:**
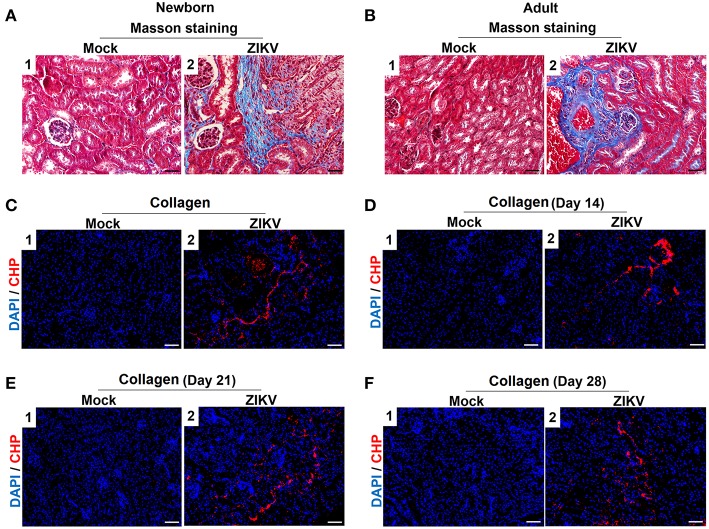
The fibrosis changes and collagen deposition in ZIKV-infected mouse kidneys. **(A,B)** Images of Masson's staining showed tubulointerstitial fibrosis (blue staining) in the kidneys of ZIKV-infected newborn mice **(A)** and adult mice **(B)** at 14 days post-infection, scale bar 40 μm. **(C–F)** The kidney sections were stained with B-CHP (collagen hybridizing peptide, biotin conjugate), which were visualized using QDs-labeled streptavidin (in red) in the kidneys of ZIKV-infected newborn mice at 14 days post-infection **(C)** and adult mice at 14, 21, and 28 days post-infection **(D–F)**, scale bar 10 μm.

Furthermore, masson's trichome staining clearly demonstrated tubulointerstitial fibrosis at 14 days post-infection in the kidneys of ZIKV-infected mice (Figures 3A,B), suggesting damages of kidney parenchyma after ZIKV infection. Finally, collagenous impairment was detected by collagen hybridizing peptide (CHP) staining. Strong signals were recorded in the interstitial space around the renal tubules at 14 days post-infection in the kidneys of ZIKV-infected mice (Figures 3C,D), indicating the accumulation of unfolded/degraded collagen in the infected kidneys. More importantly, the degraded collagen could persistently be detected by CHP staining in the kidneys of ZIKV-infected adult mice at 21 and 28 days post-infection (Figures 3E,F), indicating a long term consequence induced by ZIKV infection in the kidneys.

### Inflammatory Response Was Involved in ZIKV-Induced Kidney Injury

**Figure 4:**
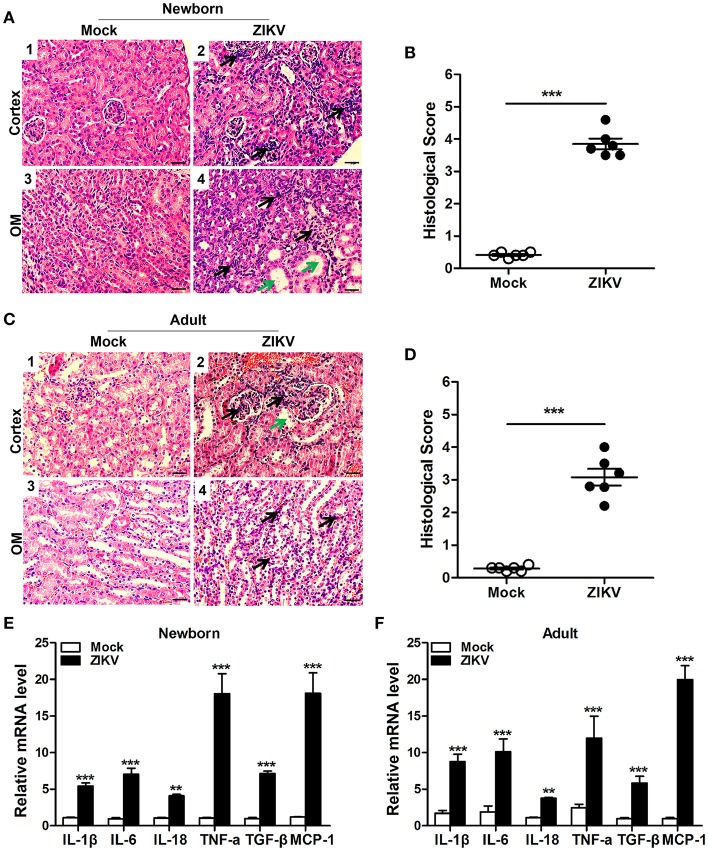
ZIKV infection increased inflammatory cell infiltration and cytokines expression in the kidneys. **(A–D)** Morphological changes and quantification of histological damage in the cortex and outer medulla (OM) of newborn **(A)** and adult **(C)** mouse kidneys at 14 days post-infection. Tissue sections were stained with hematoxylin and eosin (H&E) (scale bar: 20 μm); green arrows in the images indicated cell vacuolization; black arrows indicated inflammatory cell infiltration. **(B–D)** The histological score of newborn mice **(B)** and adult mice **(D)** were determined by tubular dilatation, vacuolization, tubular cell necrosis, loss of brush border, interstitial edema, and inflammatory cell infiltration. **(E,F)** The relative mRNA expression levels of IL-1β, IL-6, IL-18, TNF-α, TGF-β, and MCP-1 in the kidneys of mock and ZIKV-infected newborn **(E)** and adult **(F)** mice at 7 days post-infection were determined by qRT-PCR. Data were presented as mean ± SEM (*n* = 6), ^**^p < 0.01, ^***^p < 0.001 vs. mock group.

We also analyzed the histopathology like inflammatory cell infiltration and renal tubular structure anomalies. H&E staining revealed the increased number of infiltrating immune cells in the kidneys of ZIKV-infected mice compared to those of mock mice in the cortex (Figures 4A1,2,C1,2) and outer medulla (OM) (Figures 4A3,4,C3,4). In addition, tubular vacuolization and brush border loss were observed in the outer region of the outer medulla (Figures 4A4,C4), which was an indication of renal tubule damage. Moreover, the histological score in the kidneys of ZIKV-infected mice was obviously higher than mice of mock group, which was determined by tubular dilatation and vacuolization, interstitial edema, brush border defect and inflammatory cell infiltration (Figures 4B,D). Furthermore, to confirm the inflammation in the kidneys of mice infected with ZIKV, we next assessed the expression levels of inflammatory factors, such as IL-1β, IL-6, IL-18, TNF-α, TGF-β, and MCP-1, in the kidney tissues. Consistent with the increased infiltration of immune cells in the kidneys, the overall expression levels of these inflammatory factors were significantly increased in ZIKV-infected mice during acute phase (Figures 4E,F). Therefore, our data suggested that ZIKV infection-induced inflammatory cell infiltration and inflammation might be involved in kidney dysfunction.

### ZIKV Infection Triggered NLRP3 Inflammasome Activation and IL-1β Production

**Figure 5:**
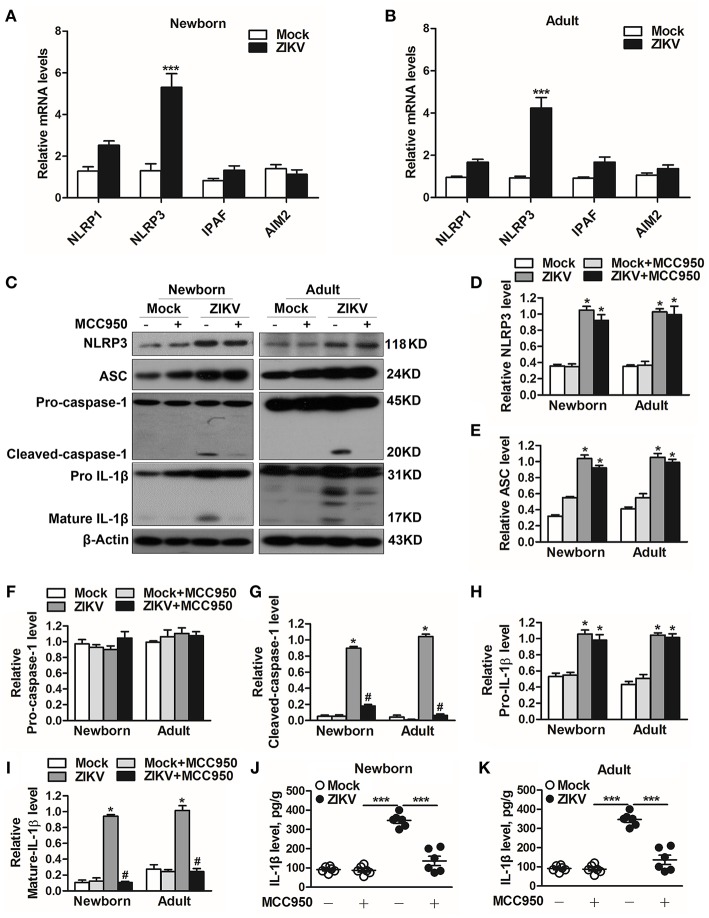
ZIKV infection induced NLRP3 inflammasome activation in the kidneys. **(A,B)** The relative mRNA levels (vs. mock group) of NLRP1, NLRP3, IPAF, and AIM2 in the kidneys of mock and ZIKV-infected newborn **(A)** and adult **(B)** mice at 7 days post-infection were detected by qRT-PCR. **(C–I)** Expression levels of NLRP3 **(D)**, ASC **(E)**, pro-caspase-1 **(F)**, cleaved-caspase-1 **(G)**, pro-IL-1β **(H)**, and mature IL-1β **(I)** in the kidneys of newborn and adult mice at 7 days post-infection were assessed by western blot. **(J,K)** Examination of mature IL-1β release in the supernatants of mouse kidneys at 7 days post-infection (*n* = 6 per group) were determined by an enzyme-linked immunoadsorbent assay (ELISA). Data were presented as mean ± SEM (*n* = 6), ^*^p < 0.05 vs. mock group; #*p* < 0.05 vs. ZIKV group (^***^p < 0.001 vs. mock group or ZIKV+MCC950 group in Figure J and K).

Previous studies have reported that the activation of NLRP3 inflammasome triggered the host inflammatory responds in some renal diseases (16, 29). It's possible to deduce that inflammasome may be involved in ZIKV-induced AKI. Therefore, we further assessed the changed expression of different inflammasome component in ZIKV-infected mouse kidneys. Obviously, the mRNA expression level of NLRP3 was significantly increased in the ZIKV-infected mouse kidneys, but not NLRP1, IPAF, or AIM2 (Figures 5A,B). Next we determined the protein expression levels of NLRP3 inflammasome activation, including NLRP3, ASC, caspase-1, and IL-1β in the kidneys of mice infected with ZIKV. The results showed that the expression levels of NLRP3, ASC, cleaved-caspase-1, pro-IL-1β and mature IL-1β were significantly up-regulated in the kidneys of both newborn and adult mice infected with ZIKV compared with those of mock mice (Figures 5C–I). Whereas, the protein expression levels of cleaved-caspase-1 and mature IL-1β were significantly decreased in the kidneys of mice treated with MCC950 (Figures 5G,I), and the release of IL-1β in the supernatant of mouse kidney was also down-regulated (Figures 5J,K), indicating that the inhibition of NLRP3 inflammsome with MCC950 suppressed ZIKV infection-induced NLRP3 inflammasome activation. More importantly, inhibition of the NLRP3 inflammasome alleviated ZIKV infection-induced kidney injury, which was indicated by the decreased expression of AKI-related biomarkers in the mouse kidneys (Supplemental Figure 2). Overall, we concluded that ZIKV infection induced AKI by triggering NLRP3 inflammasome activation and inflammatory response in the kidneys.

### IL-1β Suppressed Aquaporins Expression and Led to Water Re-Absorption Disorder

**Figure 6:**
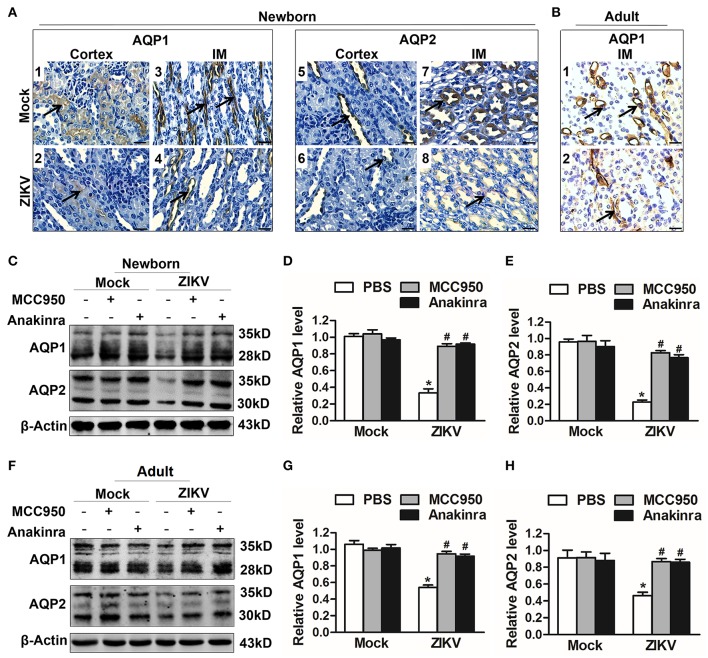
ZIKV-induced IL-1β decreased the expression of aquaporins in renal cells. **(A,B)** Immunohistochemistry analysis of kidney sections showed comparable low immunoreactivity in the AQP1 and AQP2 labeling in both cortex and inner medulla (IM) of kidneys in newborn mice **(A)** and adult mice **(B)** at 7 days post-infection. The black arrows depicted AQP1and AQP2 expression. Scale bars, 40 μm. **(C–H)** The expression of AQP1 and AQP2 in the kidneys of newborn mice **(C–E)** and adult mice **(F–H)** were assessed by western blot at 7 days post-infection. The statistical graphs were shown as the mean ± SEM; *N* = 6 mice/group, ^*^p < 0.05 vs. mock group; #*p* < 0.05 vs. ZIKV group.

Studies reported that the constant increase of IL-1β reduces AQP2 expression, indicating that inflammatory cytokines may be involved in the abnormal expression of aquaporins (21). Notably, the activation of NLRP3 inflammasome significantly promoted the release of IL-1β in the supernatants of mouse kidneys at 7 days post-infection (Figures 5J,K). In addition, the immunohistochemistry analysis showed exactly decreased expression levels of AQP1 and AQP2 proteins in the kidneys of both newborn (Figure 6A) and adult mice (Figure 6B) infected with ZIKV. To explore whether the NLRP3 inflammasome/IL-β was involved in the reduction of AQP1 and AQP2 expression in the kidneys, mice were treated with MCC950 and Anakinra during ZIKV infection, which are NLRP3 inflammasome inhibitor and IL-1β receptor antagonist, respectively. Our data showed that the expression of AQP1 and AQP2 were significantly reduced in the kidney of newborn (Figures 6C–E) and adult mice (Figures 6F–H) infected with ZIKV, but the reduction could be restored by MCC950 and Anakinra treatment (Figures 6C–H). To further confirm whether IL-1β could directly inhibit the AQP1/AQP2 expression, primary murine renal epithelial cells were treated with recombinant IL-1β for 24 h. Our data showed significant decreased expression levels of AQP1 and AQP2 in the cells treated with IL-1β (Supplemental Figures 3A,B). Consequently, these results suggested that ZIKV-induced IL-1β production inhibited AQP1 and AQP2 expression and probably compromised the ability of the kidneys to maintain water in the tubular segments, which was consistent with severe tubular injuries observed in ZIKV-infected mice.

### The Inhibition of Bcl-2 Facilitated the Activation of NLRP3 Inflammasome

**Figure 7:**
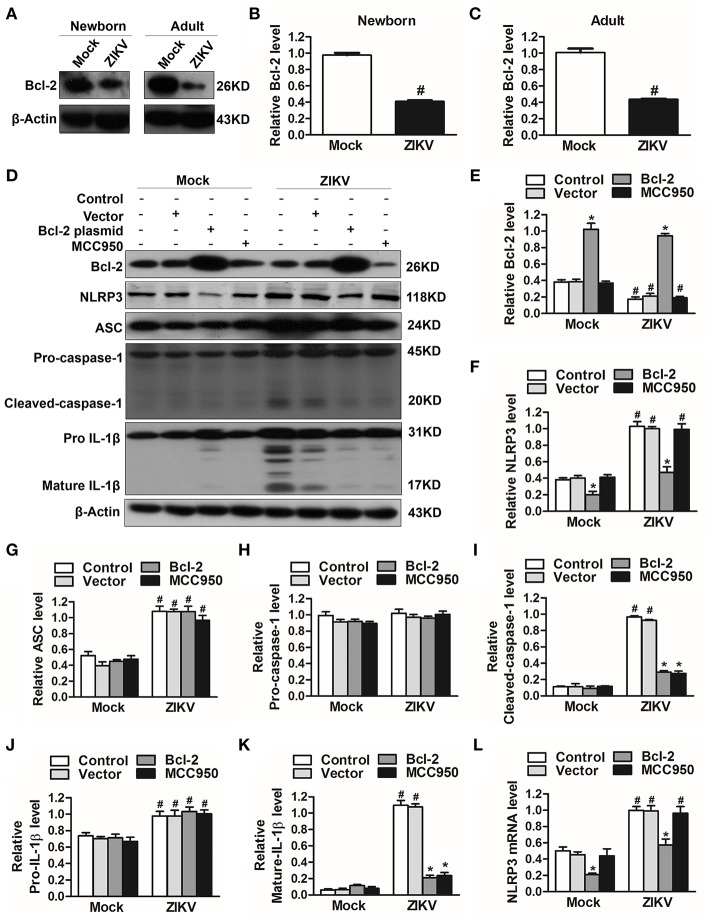
Overexpression of Bcl-2 attenuated ZIKV-induced NLRP3 inflammasome activation in HK-2 cells. **(A–C)** Expression level of Bcl-2 in the kidneys of newborn **(A,B)** and adult mice **(A,C)** at 7 days post-infection were assessed by western blot. **(D–K)** HK-2 cells were transfected with control vector or pSG5-FLAG-Bcl-2 plasmid (2 μg/ml) for 24 h or pre-treated with MCC950 (10 μM) for 30 min followed by incubation with PBS (mock group) or ZIKV (MOI = 2), and cultured with complete medium in presence of MCC950 or PBS at 37°C for 24 h. The protein expression levels of Bcl-2, NLRP3, ASC, pro-caspase-1, cleaved-caspase-1, pro-IL-1β, and mature IL-1β protein were assessed by western blot. **(L)** The mRNA expression level of NLRP3 was detected by qRT-PCR. Data were presented as mean ± SEM, ^*^p < 0.05 vs. Control group; #*p* < 0.05 vs. mock group.

To investigate which host factor was involved in the activation of NLRP3 inflammasome, we further analyzed the expression level of Bcl-2, which was reported to be a novel negative regulator of inflammasome activation (25). In our study, the expression of Bcl-2 was decreased in the kidneys of ZIKV-infected mice (Figures 7A–C). To investigate the role of Bcl-2 in the regulation of NLRP3 inflammasome activation, we constructed the Bcl-2 plasmid to overexpress Bcl-2 into HK-2 cells. ZIKV infection reduced the expression level of Bcl-2 in HK-2 cells, whereas cells transfected with Bcl-2 plasmid significantly enhanced Bcl-2 expression in both mock or ZIKV-infected HK-2 cells, which confirmed the efficiency of the protocol of Bcl-2 overexpression (Figures 7D,E). The NLRP3 inflammasome inhibitor MCC950 served as a positive control in these experiments. ZIKV infection activated NLRP3 inflammasome in HK-2 cells, which indicated by the increased expression level of NLRP3, ASC, cleaved-caspase-1, pro-IL-1β and mature IL-1β (Figures 7D, F–K). The results showed that the expression of NLRP3 was not affected by MCC950 treatment, but was down-regulated by Bcl-2 overexpression in both mock and ZIKV-infected HK-2 cells, suggesting that Bcl-2 inhibited the NLRP3 priming (Figures 7D,F). Both Bcl-2 overexpression and MCC950 treatment attenuated the cleavage of caspase-1 and IL-1β in ZIKV-infected HK-2 cells (Figures 7D,I,K), indicating that Bcl-2 overexpression attenuated ZIKV-induced NLRP3 inflammasome activation. More importantly, Bcl-2 overexpression not only inhibited the NLRP3 protein expression level but also the mRNA expression level in both mock and ZIKV-infected mice (Figures 7F,L). Therefore, these findings indicated that ZIKV infection induced NLRP3 inflammasome activation by inhibiting the expression of Bcl-2.

### Bcl-2 Suppressed Renal Apoptosis by Down-Regulating the PARP/Caspase-3 Apoptotic Signaling Pathway

**Figure 8:**
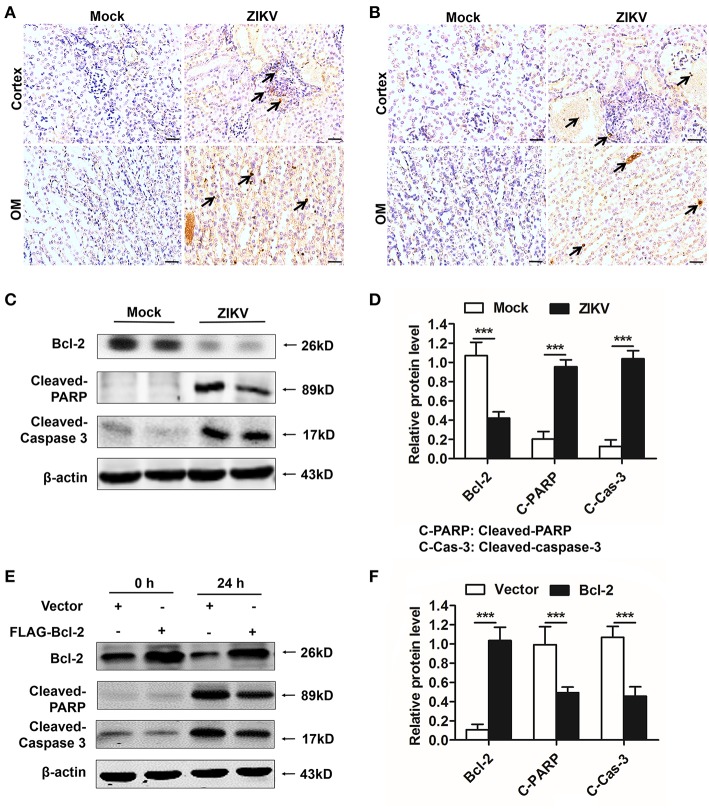
Overexpression of Bcl-2 down-regulated the expression of cleaved caspase-3 and PARP in ZIKV-infected renal cells. **(A,B)** Cell apoptosis was tested in the kidneys of ZIKV- infected newborn **(A)** and adult mice **(B)** at 14 days post-infection using a TUNEL assay kit. Positive apoptotic cells detected by the DAB reagent had a brownish-yellow nucleus, which indicated by black arrows. Scale bars, 20 μm. **(C,D)** The expression of Bcl-2, cleaved caspase-3, and cleaved PARP in the kidneys of newborn mice were assessed by western blot. **(E,F)** HK-2 cells were transfected with control vector or pSG5-FLAG-Bcl-2 plasmid (2 μg/ml) for 24 h followed by incubation with PBS (mock group) or ZIKV (MOI = 2) for 24 h. Expression levels of FLAG, cleaved caspase-3 and cleaved PARP protein at 0 h and 24 h post-infection were assessed by western blot. Data were presented as mean ± SEM, (^***^p < 0.001 vs. mock or vector group).

As an anti-apoptotic protein, Bcl-2 is identified as a negative regulator of apoptotic signaling pathway (30). Firstly, to confirm the apoptosis in the kidney of mice infected with ZIKV, we measured apoptosis using TUNEL staining. Consistent with a significant kidney injury, marked TUNEL staining confirmed the presence of tubular cell apoptosis in the renal cortex and outer medulla (OM) of ZIKV-infected newborn (Figure 8A) and adult mice (Figure 8B). Furthermore, enhanced expression levels of cleaved caspase-3 and PARP (poly ADP-ribose polymerase) (Figures 8C,D) and reduced levels of Bcl-2 were observed in the kidneys of ZIKV-infected mice (Figures 8C,D). These results suggested that ZIKV infection induced renal apoptosis by the down-regulated expression of Bcl-2 and the up-regulated expression of cleaved caspase-3 and PARP. To further elucidate the role of Bcl-2 in the regulation of ZIKV-induced apoptosis, we overexpressed Bcl-2 in HK-2 cells using its plasmid. In our results, overexpression of Bcl-2 significantly suppressed the expression levels of cleaved caspase-3 and PARP in HK-2 cells infected with ZIKV (Figures 8E,F). Therefore, our data suggest that Bcl-2 attenuated renal apoptosis by down-regulating caspase-3/PARP apoptotic signaling pathway.

### Bcl-2 Played a Protective Role in ZIKV-Induced Renal Cell Injury

**Figure 9:**
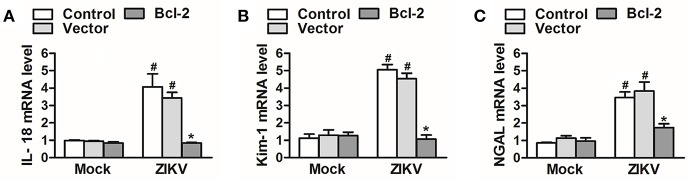
Overexpression of Bcl-2 attenuated renal cell injury induced by ZIKV infection. **(A–C)** The relative mRNA levels of IL-18, NGAL, and Kim-1 were determined by qRT-PCR. Data were presented as mean ± SEM, ^*^p < 0.05 vs. Control group; #*p* < 0.05 vs. mock group.

Since Bcl-2 attenuated the activation of NLRP3 inflammasome and apoptosis of renal cells, we further confirm whether Bcl-2 reduced renal cell injury induced by ZIKV infection. We detected the expression levels of AKI-related-markers IL-18, NGAL, and kim-1 in Bcl-2-overexpressed HK-2 cells. Our results showed that overexpression of Bcl-2 extremely decreased IL-18, NGAL, and kim-1 mRNA expression levels in HK-2 cells infected with ZIKV (Figures 9A–C), indicating the protection role of Bcl-2 on renal cell injury.

## Discussion

**Figure 10:**
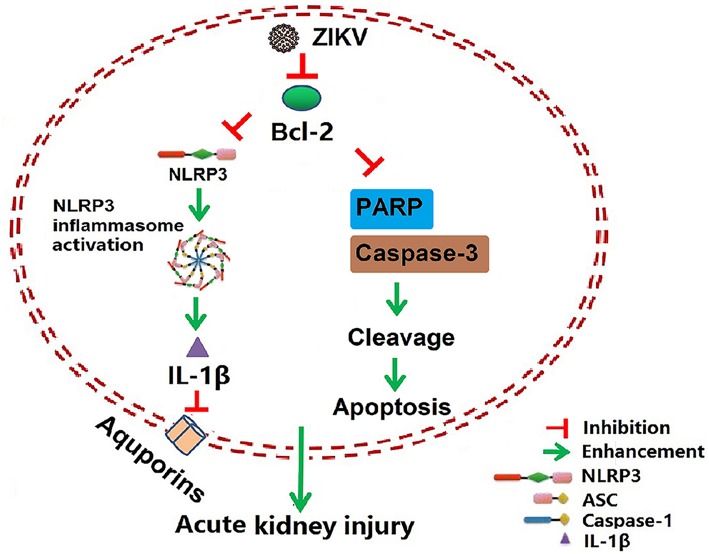
Schematic of the mechanism of AKI induced by ZIKV infection. ZIKV infection promotes NLRP3 inflammasome activation and IL-1β production in the kidney by suppressing the expression of Bcl-2. IL-1β attenuates the expression of aquaporins, thus leading to water re-absorption disorder and renal cell injury. In addition, ZIKV infection increases PARP and caspase-3-mediated renal apoptosis via inhibiting Bcl-2, which further enhances the kidney injury. Overall, ZIKV infection induces acute kidney injury through activating NLRP3 inflammasome via suppressing Bcl-2.

Isolation of ZIKV from various fluids and organs has been reported. *In vitro* studies have shown that ZIKV can infect glomerular cells and renal proximal tubular epithelial cells (9, 10). However, how ZIKV infection affects renal function *in vivo* and the underlying mechanism remain unknown. Here, we reported the Asian strain of ZIKV (ZG01) isolated from the urine of a Chinese patient could infect renal epithelial cells. The ZIKV persisted for long periods in the kidney and urine, thus inducing kidney injury in newborn and adult mice. ZIKV infection suppressed Bcl-2 expression and induced excessive inflammatory responses by activating the NLRP3 inflammasome, which subsequently led to kidney injury shown by impaired glomerular filtration and tubular re-absorption ability. Bcl-2 overexpression in HK-2 cells inhibited ZIKV-induced NLRP3 inflammasome activation and renal apoptosis, thus reducing renal cell injury. Therefore, our work identified a novel role of ZIKV infection in inducing AKI by activating NLRP3 inflammasome via suppressing Bcl-2 (Figure 10). Our study is the first to clearly link ZIKV infection to kidney injury and elucidate the underlying molecular mechanisms.

We demonstrated that mice infected with ZIKV develop AKI according to the increase levels of following indicated biomarkers. Scr is a metabolite of creatine and excreted unchanged into urine (31). The high level of Scr concentration accumulated in the serum reflects an impaired glomerular filtration in ZIKV-infected mice in our study. NGAL is highly up-regulated in mRNA level after kidney injury (31) and is detectable as early as 3 h after the injury (32). The elevation can persist up to 7 days after the initial injury when the injury is severe. Therefore, it is a widely acceptable biomarker of tubular injuries, especially in the early stage of kidney injuries (33). Kim-1 is also an ideal biomarker of AKI because of its low level or absence expression in the normal kidney, and it will rapidly increase after kidney injury (34, 35). Kim-1 plays an important role in kidney recovery and tubular regeneration, as it reflects the tubular injury (36). In our study, the increased expression levels of NGAL and Kim-1 in mouse kidneys during ZIKV infection indicated AKI occurred in ZIKV-infected mice. In addition, the tubular injury was supported by increased urine output and thus also indicated decreased tubular re-absorption. Consistent with this phenomenon, the protein expression levels of renal AQP1 and AQP2 were markedly reduced in mice infected with ZIKV. Expressed in the proximal tubule and descending thin limb, AQP1 contributes to 80% of the total water re-absorption (37). AQP2 in collecting tubule plays an important role in water re-absorption before urine is excreted (38, 39). The reduced expression of these proteins contribute to the increased urine output in mice infected with ZIKV.

Local and systemic inflammation can negatively influence kidney function including water re-absorption, especially in the occurrence of AKI during pathogen infection or endotoxemia (40). The inflammatory response of the kidney is usually characterized by a large infiltration of inflammatory cells. In our study, H&E staining showed extensive immune cell infiltration and inflammatory cytokines release in the kidneys of mice infected with ZIKV, suggesting the inflammatory responses occurred. Moreover, pronounced tubulointerstitial fibrosis recognized as a key determinant of progressive kidney disease (41), was found in infected kidneys. The increased tubulointerstitial fibrosis observed in ZIKV-infected mice could be attributed to the tissue damage induced by the inflammatory response. Therefore, it's possible that the injury observed in the mouse kidney was due to the uncontrolled infiltration of immune cells triggered by the replication of ZIKV. Consistently, the extremely increased level of cytokines (e.g., TNF-α, TGF-β, IL-1β, and IL-18) indicated the activation of NF-κB signaling in ZIKV-infected mouse kidneys, which was considered as Signal 1 for inflammasome activation.

The activation of NLRP3 inflammasome is involved in various inflammatory diseases and plays an important role in the development of AKI (16). NLRP3 inflammasome is a multi-protein complex and consists of NLRP3, ASC, and pro-caspase-1, which mediates the activation of caspase-1 and the secretion of mature IL-1β and IL-18 (42). IL-1β is a highly potent pro-inflammatory factor that induces vasodilation and attracts granulocytes to the inflamed tissues, therefore facilitating severe inflammatory disease development (43). Recent studies have shown that NLRP3-mediated IL-1β secretion directly inhibits AQP2 expression in the primary collecting duct cells (21), which is consistent with our results. We found that both of ZIKV infection and recombinant IL-1β treatment resulted in a significant decrease expression of AQP1 and AQP2 in primary murine renal epithelial cells thus inducing water re-absorption disorder, which indicated that inflammasome was involved in ZIKV-induced renal dysfunction.

Many viruses infection can induce the secretion of IL-1β to promote the development of severe inflammatory disease by activating NLRP3 inflammasome, including DENV, JEV, influenza virus, and human immunodeficiency virus type 1 (HIV) (44–48). Recently studies have reported that ZIKV-NS5 directly binds to NLRP3 and facilitates NLRP3 inflammasome assembly in ZIKV-infected tissues, but their studies focus on immune cells, so that the related host factors involved in the regulation of NLRP3 inflammasome need further study (49, 50). In our study, NLRP3 inflammasome could be activated by ZIKV infection in non-immunocytes renal epithelia cells HK-2. The NLRP3 inflammasome activation promoted the cleavage of caspase-1 and the release of mature IL-1β, thus resulting in excessive inflammatory responds and renal damage.

NLRP3 inflammasome activation is widely considered to induce pyropotic cell death and has been shown to contribute to the infammatory response of various models of kidney injury (16, 51). It is reported that the inflammasome-activated caspase-1 is involved in the apoptosis process, followed by gasdermin D dependent secondary necrosis/pyroptosis (52). Furthermore, the gasdermin pores permeabilize mitochondria to enhance caspase-3 activation, which indicates the crosstalk between pyroptosis and apoptosis signaling pathways (53). Actually, the activation of NLRP3 inflammasome is reported to be associated with cell apoptosis in contrast media-induced AKI. The disruption of NLRP3 inflammasome alleviates the expression and activation of apoptosis associated proteins (54). In our study, the inhibition of NLRP3 inflammasome activation slightly suppressed ZIKV infection-induced cell death (Supplemental Figure 4A), indicating that NLRP3 inflammasome activation contributed to cell death, but it was not enough to eliminate the ZIKV infection-induced cell death. More importantly, Bcl-2 played a negative role in both ZIKV infection-induced NLRP3 inflammasome activation and apoptosis by down-regulating the NLRP3 priming and the expression of pro-apoptotic proteins, respectively. Therefore, we supposed that Bcl-2 may be a novel link between NLRP3 and apoptosis signaling pathway. Bcl-2 can suppress the activity of caspase-1 by directly binding to NLRP1, which fortifies the possibility of Bcl-2 in regulating the NLRP3 inflammasome activation (24, 25). Whereas, whether Bcl-2 directly binding to NLRP3 or inhibiting its transcriptional translation need to be further studied. On the other hand, as an anti-apoptotic protein, Bcl-2 plays a central role in mitochondria-mediated apoptosis, which down-regulates the activation of apoptotic signaling pathway by attenuating the expression of pro-apoptotic proteins (55, 56). This is consistent with our findings, over-expression of Bcl-2 suppresses renal apoptosis and AKI-related-markers IL-18, NGAL, and Kim-1 expression in ZIKV-infected HK-2 cells, thus preventing the renal cell injury.

Over the present study, we performed all the experiments using the Asian stain (ZG01) of ZIKV, which was isolated from the patient's urine sample by our lab. Whether the African strain causes renal disease or the infectious virus sheds in urine remain unknown. Actually, we established the ZIKV-infected mouse model by intraperitoneally injecting African strain MR766 in other ZIKV studies. Mice showed more susceptible to MR766 infection than Asian stain ZG01 infection. In addition, viral RNA of MR766 could also be detected in the urine of mice (data not shown). HK-2 cells was more permissive to MR766 infection than to ZG01 infection (Supplemental Figure 5), which was consistent with Jian' study (10). In their study, MR766 induced more caspase-3-mediated renal cell apoptosis, suggesting that the capacity of MR766 to cause renal abnormalities and the strain-specific pathogenesis profiles should be taken into account in future studies.

In summary, our findings illustrated the mechanism of ZIKV infection induced AKI through activating NLRP3 inflammasome via suppressing Bcl-2. The release of mature IL-1β triggered by NLRP3 inflammasome could directly down-regulate the expression of aquaporins, thus leading to re-absorption disorder in renal cells. Bcl-2 overexpression attenuated NLRP3 inflammasome activation and renal cell injury by inhibiting both mRNA and protein expression level of NLRP3. Our results were observed exclusively in mouse models, and further studies on clinical patients were therefore necessary to be performed to confirm our finding. Overall, our findings uncovered a link between ZIKV and kidney disease and identified Bcl-2 as a potential target in the intervention of ZIKV-induced AKI.

## Data Availability

The raw data supporting the conclusions of this manuscript will be made available by the authors, without undue reservation, to any qualified researcher.

## Ethics Statement

This study was carried out in accordance with the recommendations of the National Institutes of Health Guide, the SYSU Institutional Animal Care and Use Committee (SYSU IACUC). The protocol was approved by the SYSU IACUC (ethics reference number: 2016-158).

## Author Contributions

TL and MW wrote the manuscript. XH, HZho, CL, and TL designed experiments. TL, LT, HT, and DF performed experiments and analyzed data. WW, JP, SG, Y-PL, XZ, HZha, YL, and MW provided scientific expertise. XH supervised the project.

### Conflict of Interest Statement

The authors declare that the research was conducted in the absence of any commercial or financial relationships that could be construed as a potential conflict of interest.
