# Supplementary material for: Zika Virus Infection Induces Acute Kidney Injury Through Activating NLRP3 Inflammasome Via Suppressing Bcl-2
Source: Front Immunol. 2019 Aug 14;10:1925. doi: 10.3389/fimmu.2019.01925 (PMC6702322; doi:10.3389/fimmu.2019.01925)
Supplement: Supplementary file 1 [file Data_Sheet_1.pdf]

Supplemental Figures and Legends

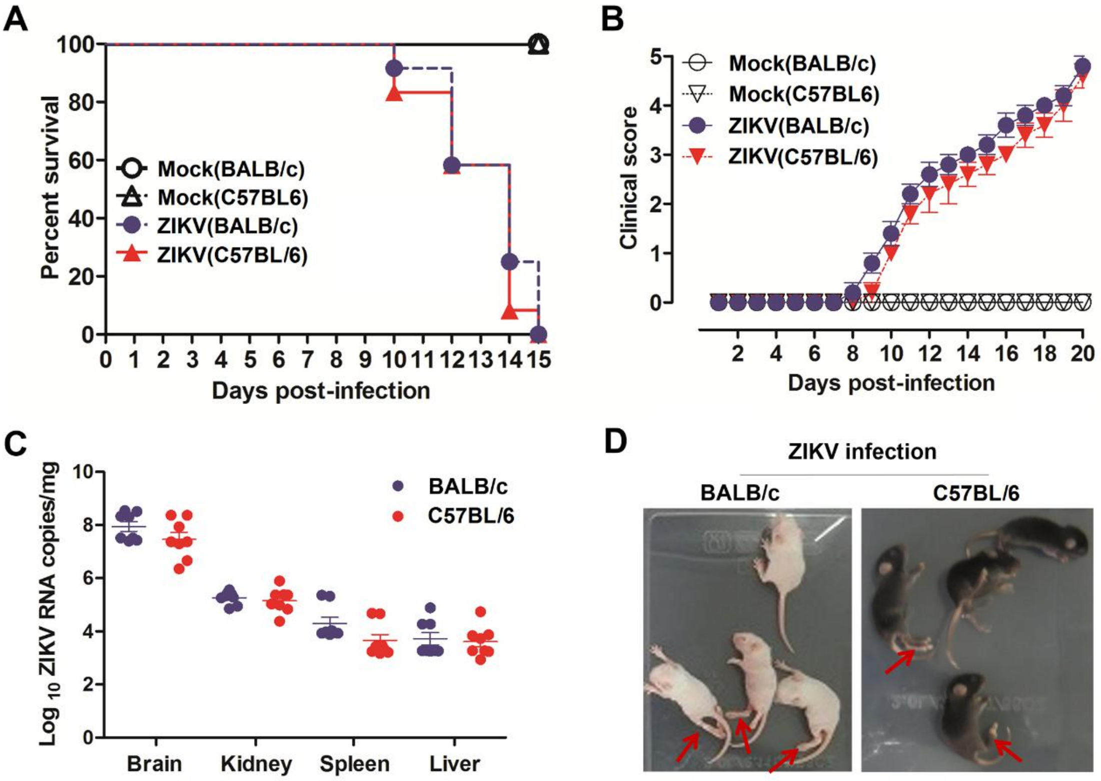

Supplemental Figure 1. Establishment of ZIKV-infected BALB/c and C57BL/6 newborn mouse model. One- or two-day-old mice were infected with ZIKV (10^5 PFU) by intraperitoneal injection. **(A)** Lethality was monitored for 15 days in the BALB/c and C57BL/6 newborn mice infected with ZIKV. **(B)** The clinical scores of uninfected (mock) and ZIKV-infected BALB/c or C57BL/6 mice were recorded over 15 days. Clinical scores were scored as follows: 0: healthy, 1: body weight loss, 2: unsteady gait, 3: severe ataxia, 4: paralysis, and 5: death. **(C)** Viral RNA copies in whole brain, kidney, spleen, and liver tissues were determined by qRT-PCR analysis at 10 days post-infection. **(D)** Macroscopic photos revealed the severe paralysis in ZIKV-infected BALB/c and C57BL/6 newborn mice at 10 days post-infection, the red arrows indicated the paralysis legs. N = 6 mice/group.

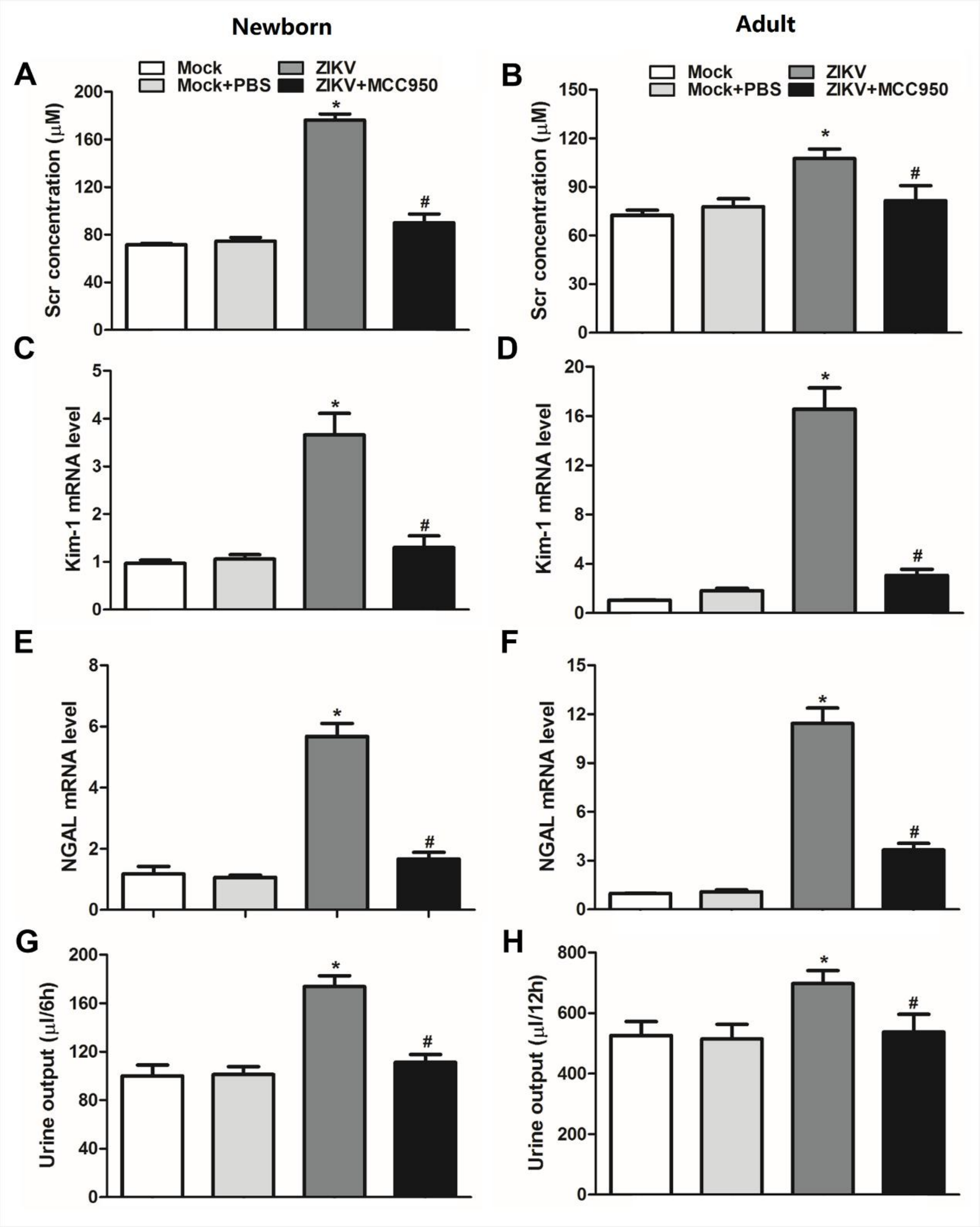

Supplemental Figure 2. The inhibition of NLRP3 inflammasome attenuated ZIKV-induced acute kidney injury in mice. Serum creatinine (Scr) concentration was tested in newborn mice **(A)** at 10 days post-infection and in adult mice **(B)** at 7 days post-infection using a creatinine assay kit. **(C-F)** The relative mRNA levels of KIM-1 **(C-D)** and NGAL **(E-F)** were determined in the kidneys of newborn mice **(E)**

at 10 days post-infection and adult mice **(F)** at 7 days post-infection by qRT-PCR. **(G-H)** Urine output was measured for 6 h in newborn mice **(G)** and 12 h for adult mice **(H)**. N = 6 mice/group. (*p <0.05 vs mock group; #p <0.05 vs ZIKV group).

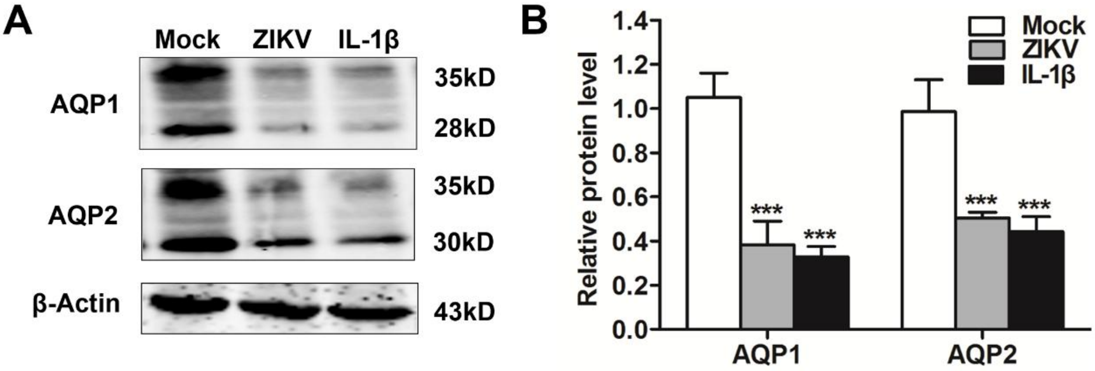

Supplemental Figure 3. IL-1β decreased the expression of aquaporins in renal cells. (A-B) The primary murine renal epithelial cells were obtained from 7-day-old newborn mice and incubated with ZIKV (MOI=2) or treated with IL-1β (5 ng/ml) for 24 h. Then the expression of AQP1 and AQP2 in the mock or ZIKV-infected or IL-1β treated renal cells were assessed by western blot. (***p < 0.001 vs. mock group).

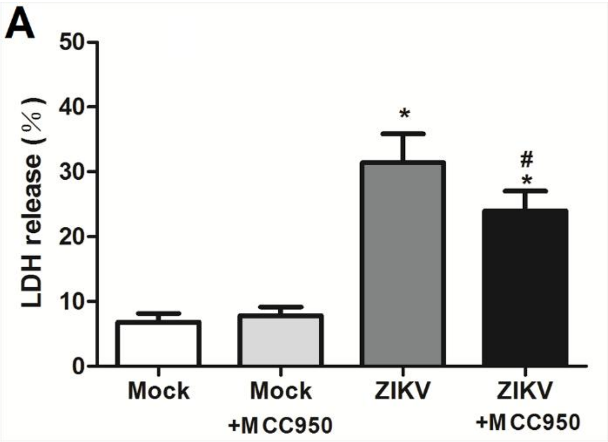

Supplemental Figure 4. The inhibition of NLRP3 inflammasome activation slightly suppressed ZIKV infection-induced cell death. HK-2 cells were pre-treated with MCC950 (10 μM) for 30min followed by incubation with PBS (mock group) or

ZIKV (MOI=2), and cultured with complete medium in presence of MCC950 or PBS at 37 °C for 24 h. **(A)** Cell supernatants and lysate were collected to analyze cell death by Lactate dehydrogenase (LDH) assay, using CytoTox 96 Non-Radioactive Cytotoxicity Assay Kit (Promega, Madison, WI, USA). Date were presented as mean ± SEM, **p* <0.05 vs mock group; #*p* <0.05 vs ZIKV group.

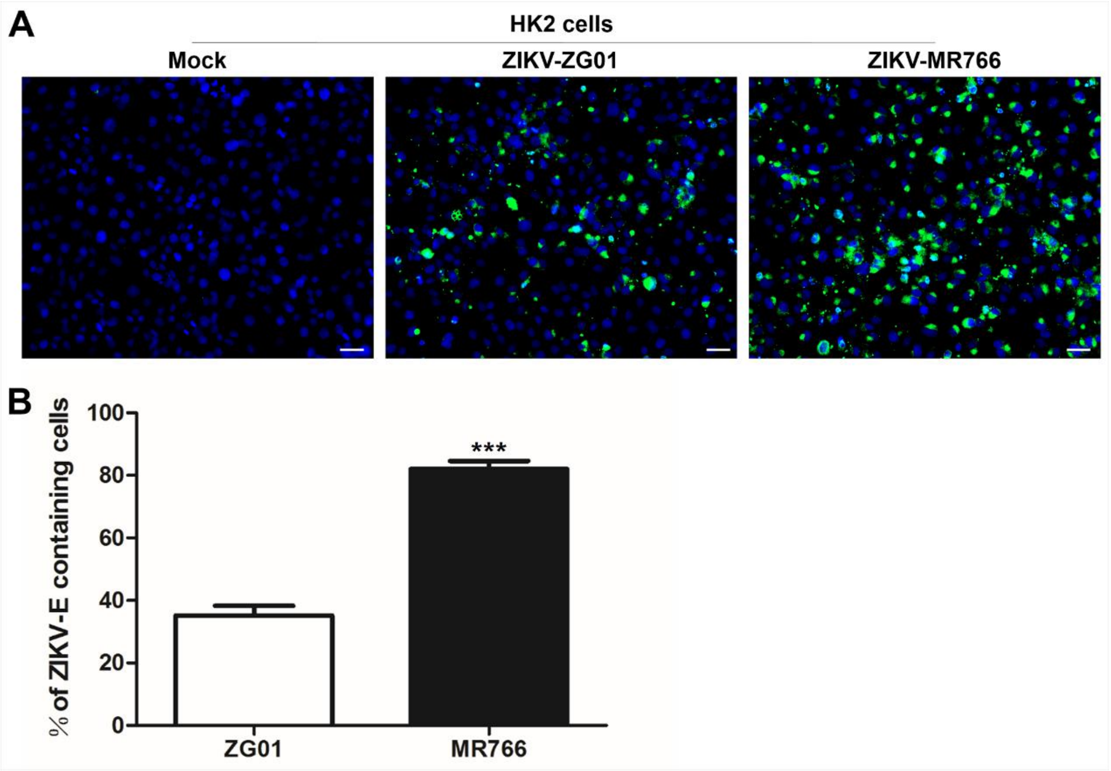

Supplemental Figure 5. HK-2 cells were more susceptible with African strain-MR766 than Asian strain-ZG01. (A-B) HK-2 cells were infected with ZG01 (Asian strain) and MR766 (African strain) (MOI=2) for 24 h, stained with DAPI to label nuclei (blue) and an antibody against ZIKV E protein (green), examined by confocal microscopy (A), Scale bars, 40 μm. The percentage of ZIKV-E protein-positive cells was calculated by image J. (****p* < 0.001 vs. mock group).
