# Supplementary material for: Zika Virus Infection Induces Acute Kidney Injury Through Activating NLRP3 Inflammasome Via Suppressing Bcl-2
Source: Front Immunol. 2019 Aug 14;10:1925. doi: 10.3389/fimmu.2019.01925 (PMC6702322; doi:10.3389/fimmu.2019.01925)
Supplement: Supplementary file 2 [file Data_Sheet_2.PDF]

Supplementary materials

Original images of western blot

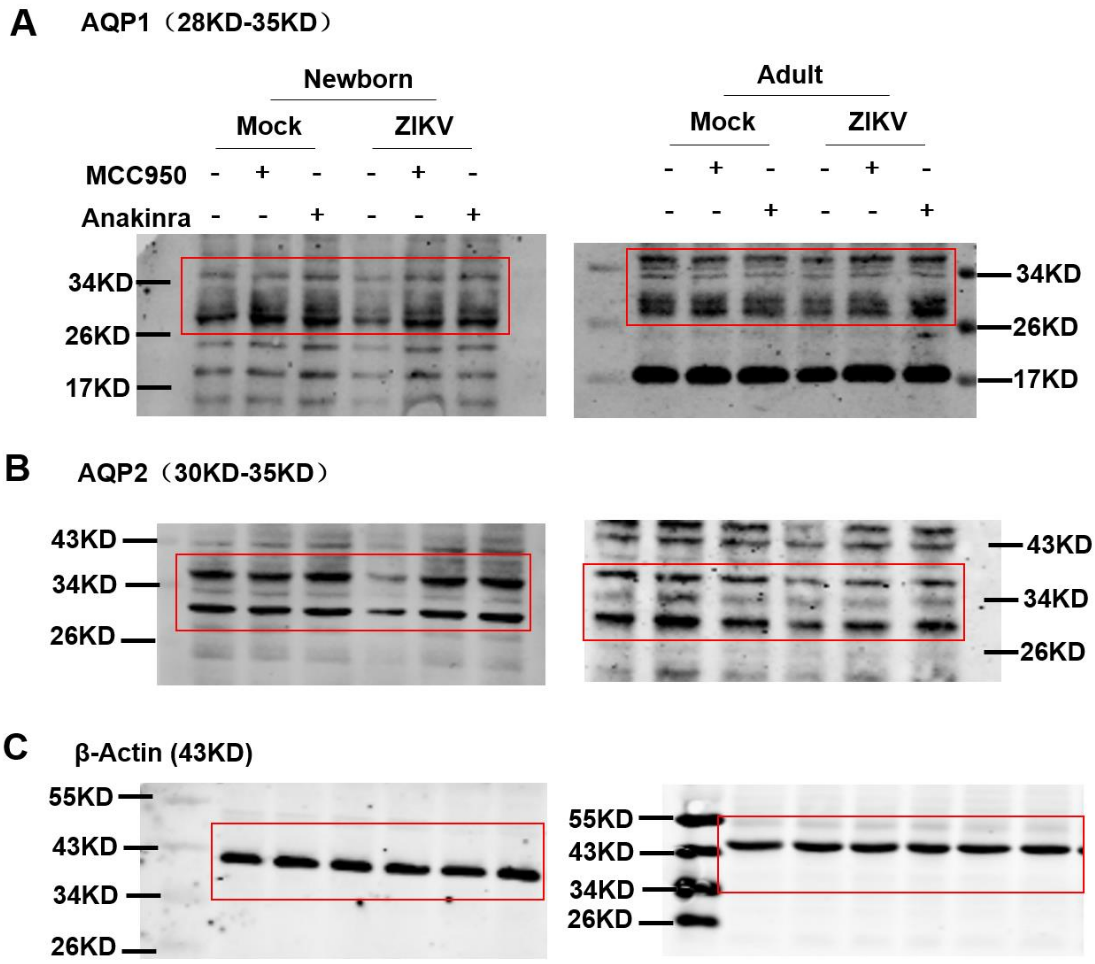

Image 1. The original images of Figure 6C and 6F in the manuscript. Impaired expression of AQP1 and AQP2 in the kidneys of ZIKV-challenged mice. **(A-C)** The expression of AQP1 **(A)**, AQP2 **(B)** and β-actin **(C)** in the kidneys of mock or ZIKV-infected newborn mice and adult mice were assessed by western blot at days 7 post-infection. The red boxes represented the position of the bands of the target proteins.

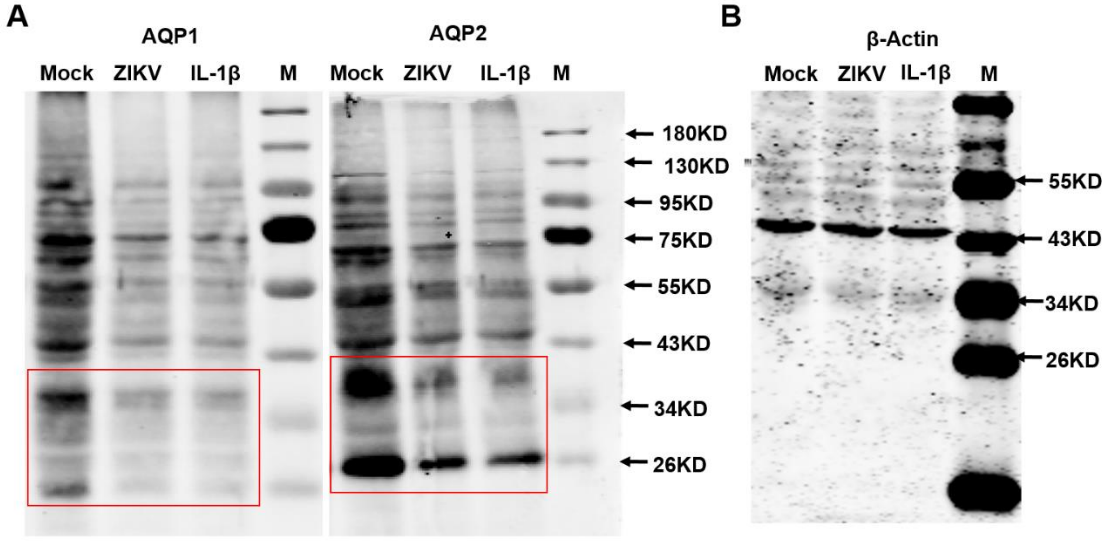

Image 2. The original images of Supplemental Figure 3A in the manuscript. IL-1β downregulated AQP1 and AQP2 expression in renal epithelia cells. **(A)** The expression of AQP1 and AQP2 in the mock or ZIKV-infected or IL-1β treated renal cells were assessed by western blot. **(B)** The expression of β-actin in the mock or ZIKV-infected or IL-1β treated renal cells were assessed by western blot. The red boxes represented the position of the bands of the target proteins.

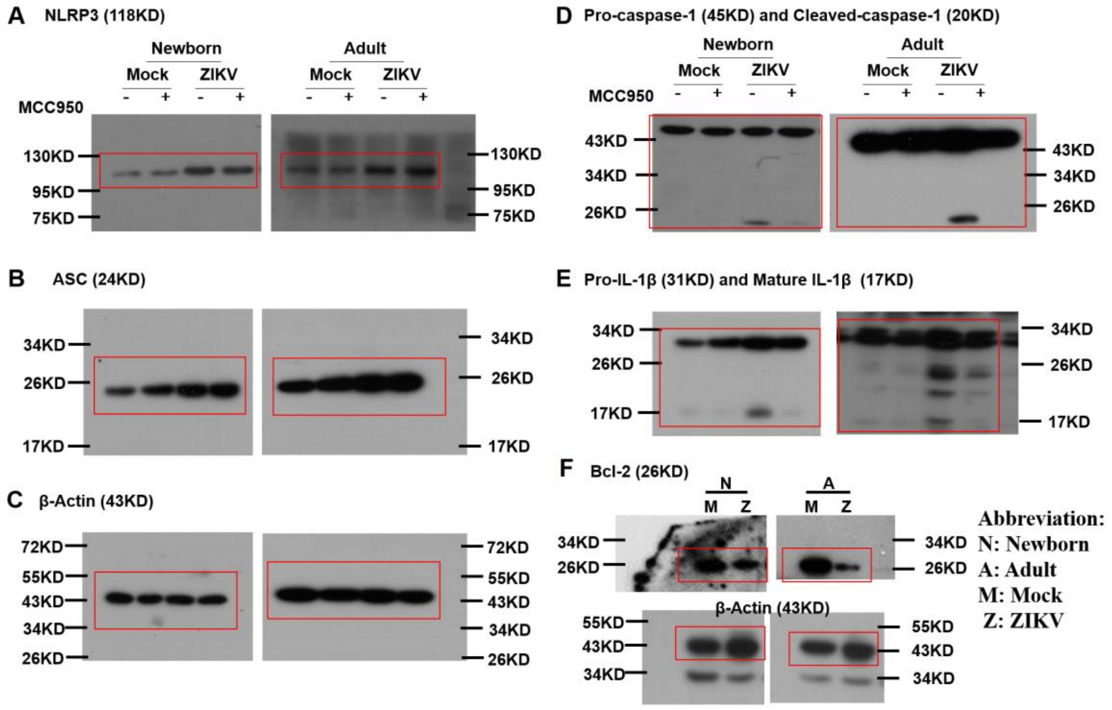

Image 3. The original images of Figure 5C and Figure 7A in the manuscript. ZIKV infection induced the activation of NLRP3 inflammasome. **(A-F)** The expression of NLRP3 **(A)**, ASC **(B)**, caspase-1 **(D)**, IL-1β **(E)** and β-actin **(C)** in the kidneys of mock or ZIKV-infected newborn mice and adult mice were assessed by western blot. **(F) The original images of Figure 7A in the manuscrip.** ZIKV infection decreased the expression of Bcl-2. The expression of Bcl-2 and β-actin in the kidneys of mock or ZIKV-infected newborn mice and adult mice were assessed by western blot. The red boxes represented the position of the bands of the target proteins.

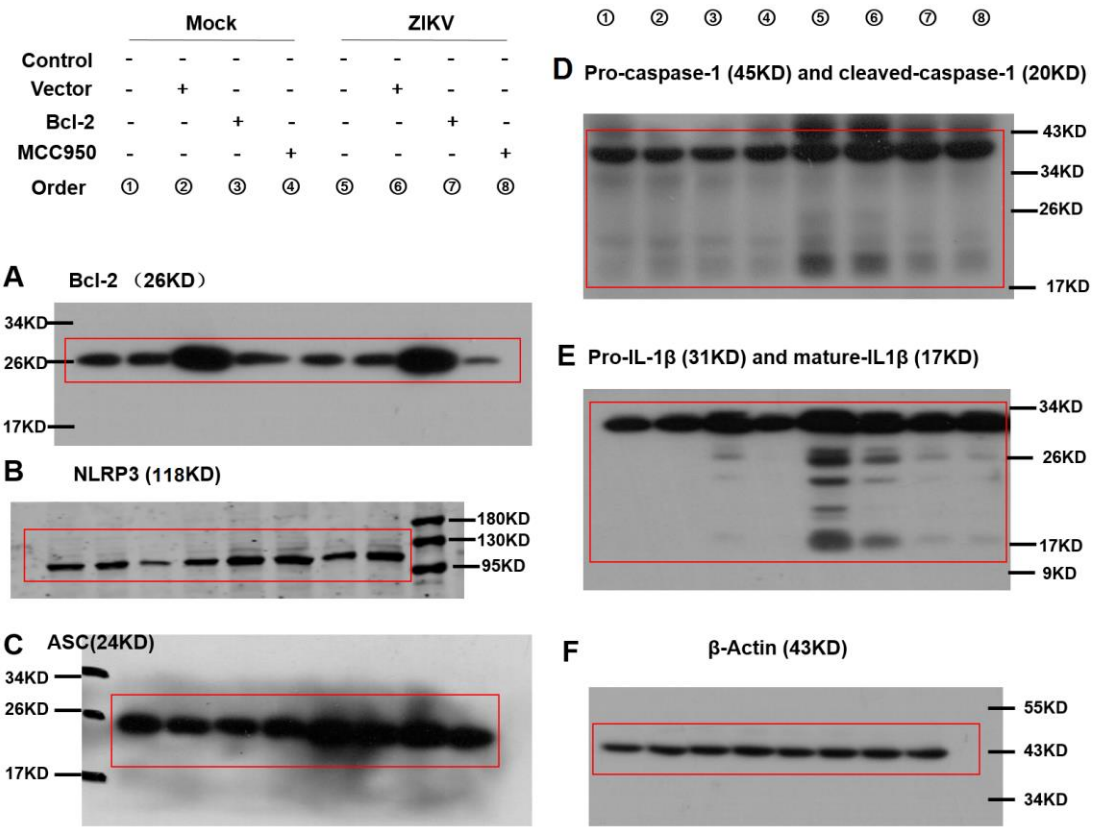

Image 4. The original images of Figure 7D in the manuscript. Overexpression of Bcl-2 attenuated the activation of NLRP3 inflammasome in HK-2 cells. **(A-F)** The expression of Bcl-2 **(A)**, NLRP3 **(B)**, ASC **(C)**, caspase-1 **(D)**, IL-1β **(E)**, β-actin **(F)** in HK-2 cells were assessed by western blot. The red boxes represented the position of the bands of the target proteins.

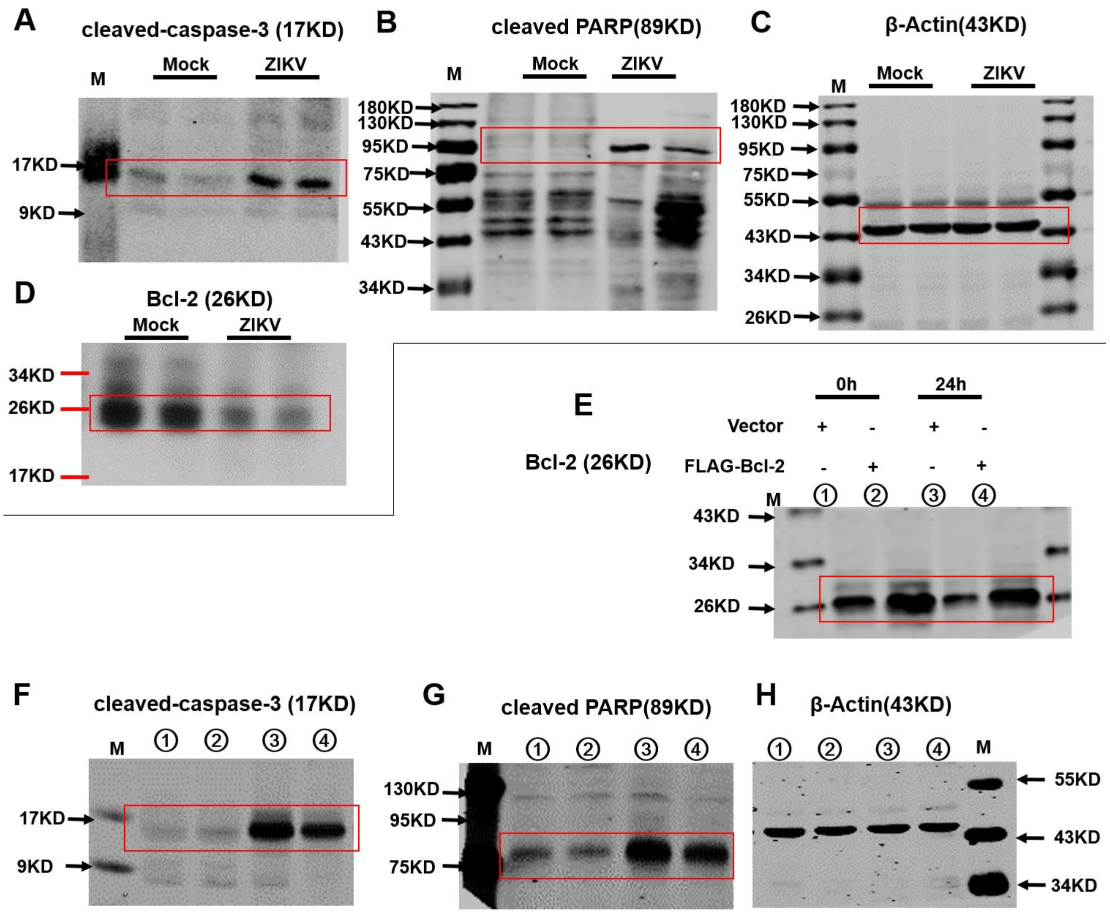

Image 5. The original images of Figure 8C and 8E in the manuscript. (A-D) ZIKV infection induced increased expression of cleaved caspase-3 and PARP and decreased expression of Bcl-2 in the kidneys. The expression of cleaved caspase-3 **(A)**, cleaved-PARP **(B)**, β-actin **(C)** and Bcl-2 **(D)** in the kidneys of mock or ZIKV-infected newborn mice and adult mice were assessed by western blot. **(E-H)** Overexpression of Bcl-2 down-regulated the expression of cleaved caspase-3 and PARP in ZIKV-infected HK-2 cells. The expression of Bcl-2 **(E)**, cleaved caspase-3 **(F)**, cleaved-PARP **(G)** and β-actin **(H)** in HK-2 cells were assessed by western blot. The red boxes represented the position of the bands of the target proteins.
